# The Protein–Protein Interaction Network of *Litopenaeus vannamei* Haemocytes

**DOI:** 10.3389/fphys.2019.00156

**Published:** 2019-02-25

**Authors:** Tong Hao, Lingxuan Zhao, Dan Wu, Bin Wang, Xin Feng, Edwin Wang, Jinsheng Sun

**Affiliations:** ^1^Tianjin Key Laboratory of Animal and Plant Resistance, College of Life Sciences, Tianjin Normal University, Tianjin, China; ^2^Cumming School of Medicine, University of Calgary, Calgary, AB, Canada

**Keywords:** protein–protein interaction network, *Litopenaeus vannamei*, haemocytes, evolutionary analyses, functional annotation

## Abstract

Protein–protein interaction networks (PINs) have been constructed in various organisms and utilized to conduct evolutionary analyses and functional predictions. *Litopenaeus vannamei* is a high-valued commercial aquaculture species with an uncharacterized interactome. With the development of RNA-seq techniques and systems biology, it is possible to obtain genome-wide transcriptional information for *L. vannamei* and construct a systematic network based on these data. In this work, based on the RNA-seq of haemocytes we constructed the first *L. vannamei* PIN including 4,858 proteins and 104,187 interactions. The PIN constructed here is the first large-scale PIN for shrimp. The confidence scores of interactions in the PIN were evaluated on the basis of sequence homology and genetic relationships. The immune-specific sub-network was extracted from global PIN, and more than a third of proteins were found in signaling pathways in the sub-network, which indicates an inseparable relationship between signaling processes and immune mechanisms. Six selected signaling pathways were constructed at different age groups based on evolutionary analyses. Furthermore, we showed that the functions of the pathways’ proteins were associated with their evolutionary history based on the evolutionary analyses combining with protein functional analyses. In addition, the functions of 1,955 unclassified proteins which were associated with 3,191 unigenes were assigned using the PIN, which account for approximately 70.3 and 44.9% of the previously unclassified proteins and unigenes in the network, respectively. The annotation of unclassified proteins and unigenes based on the PIN provides new candidates for further functional studies. The immune-specific sub-network and the pathways extracted from the PIN provide a novel information source for studying of immune mechanisms and disease resistances in shrimp.

## Introduction

Biological networks have become more commonly studied as interests grow to fulfill increasing desires for deep investigations of biological systems and mechanisms for metabolism and regulation (Wang E. et al., 2015). Protein–protein interaction networks (PINs) have been constructed in many organisms, including bacteriophages (Blasche et al., 2013), bacteria (Kim and Kim, 2009; Marchadier et al., 2011), yeast (Schwikowski et al., 2000; Ito et al., 2001; Ho et al., 2002), plants (Geisler-Lee et al., 2007; De Bodt et al., 2009; Lin et al., 2011), animals (Li et al., 2004; Guruharsha et al., 2011), and human (Rual et al., 2005). PINs have been used in various biological analysis, such as pathway identification (Navlakha et al., 2012), partition of functional modules (Chen B. et al., 2014), and annotation of novel protein functions (Zeng et al., 2008). For aquatic crustaceans, Hao et al. (2014) constructed a PIN for the Chinese mitten crab and applied it to a signaling sub-network extraction, functional prediction and evolutionary analyses. The PIN provides a blueprint for the systematic analysis of various biological activities, such as growth, immunity, and regulation.

*Litopenaeus vannamei* is an important aquatic economic animal which is belonging to Arthropoda, Crustacea, Malacostraca, Decapoda, Dendrobranchiata, Penaeidae, and Penaeus. As viral diseases have become a big threat which causes an enormous loss in the global *L. vannamei* culture industry, the investigation of immune mechanisms in *L. vannamei* for defending against viral diseases has become an important area in aquatic research. To investigate the influence of viruses on the biological processes in shrimp, differentially expressed genes after viral infections have been frequently discussed (He et al., 2005; Leu et al., 2007; Zhao et al., 2007). Protein profiles have been also investigated to find the responding mechanisms of *L. vannamei* to viral infections (Wang et al., 2007; Chen et al., 2016). Recently, several genes, such as LvHtrA2 (Peepim et al., 2016), LvVEGF1 and LvVEGF2 (Wang Z. et al., 2015), were found to have an association with viral infections. However, current researches mainly focused on the response of genes and proteins against infection, but ignoring the interactions between genes and proteins. Because the response of the *L. vannamei* immune system is a systematic process, which is not solely driven by genes independently, the systematic study of the interactions among proteins in *L. vannamei* could shed light on its immune mechanisms for against viral infections.

In this study, we constructed a PIN of *L. vannamei* haemocytes based on transcriptome sequencing data and the integration of the proteome from seven model organisms. An immune sub-network was constructed based on the global PIN, and the evolution of six signaling pathways in the immune sub-network representing a novel global view of the immune system in *L. vannamei* was investigated. In addition, the network was applied to the functional predictions for thousands of previously uncharacterized proteins and unigenes. The PIN of *L. vannamei* haemocytes is the first large-scale shrimp PIN. It provides a systematic view of the protein interactome in shrimp and other aquatic crustaceans.

## Materials and Methods

### The Protein–Protein Interaction Network Construction for *L. vannamei*

Data of the haemocyte transcriptome in *L. vannamei* were obtained from our previous studies (Xue et al., 2013). In the transcriptome sequencing, the RNA sequences were broken up into plenty of short fragments called “reads.” The “reads” were then assembled to form “contig” according to the overlap between different reads. Subsequently the “contigs” were linked together by Trinity software (Zhao et al., 2011) and eventually produces a sequence that cannot be extended at both ends, which is called “unigene.” There are totally 52,073 unigenes in *L. vannamei* haemocytes. To identify protein–protein interaction pairs in *L. vannamei*, protein sequences and their interactions of seven model organisms (*Drosophila melanogaster*, *Anopheles gambiae*, *Caenorhabditis elegans*, *Mus musculus*, *Rattus norvegicus*, *Homo sapiens*, and *Saccharomyces cerevisiae*) were first downloaded from the STRING database (Szklarczyk et al., 2017). Information from these model organisms was used as a reference for the PIN construction for *L. vannamei*. The STRING database collected the interaction information of hundreds of organisms. Based on the approximate probability that a predicted link exists between two enzymes in the same metabolic map in the KEGG database, the different ranges of confidence score of the protein–protein interactions are assigned. The unigene sequences of *L. vannamei* were aligned with the seven model organisms using BLASTX. The first aligned protein with an *E*-value below 1^∗^E-10 was considered as a homologous protein. Then, these homologous proteins and their corresponding interactions were extracted from the whole interaction dataset of the related organism to compose the model organism based protein–protein interaction sub-network. In order to get the high quality PIN, we took the interactions with the highest confidence limits 0.9 from STRING. One protein in a sub-network may be homologous to multiple unigenes. For example, the protein CG12004 in the *D. melanogaster* sub-network is homologous to two unigenes (unigene41436 and unigene41437) in *L. vannamei*. Only the interactions with both constituent proteins matching particular unigenes in *L. vannamei* were extracted. We finally got seven model organism based sub-networks altogether. We also downloaded the STRING-Uniprot ID mapping file from STRING database to label the uniprot IDs and protein names to the proteins. For the proteins which do not have Uniprot IDs and names, the STRING IDs were used.

The seven model organism based sub-networks were then integrated to construct the *L. vannamei* PIN. Both genetic relationships and the size of the sub-networks were considered in the determination of the integration order. First, the organism with the closest genetic relationship to *L. vannamei* was integrated preferentially. Second, for organisms with a similar genetic relationship and an obvious difference in data size, such as *M. musculus* and *R. norvegicus*, the sub-network with the larger size was integrated preferentially. Therefore, the final integration order was as follows: *D. melanogaster*, *A. gambiae*, *C. elegans*, *M. musculus*, *R. norvegicus*, *H. sapiens*, and *S. cerevisiae*. Network integration was performed as outlined in our previous work (Hao et al., 2014).

### Scoring of Protein–Protein Interacting Pairs

As protein–protein interaction pairs come from different model organisms, which have different genetic relationships with *L. vannamei*, the confidence scores of interaction pairs in the PIN were evaluated. The score of each interaction pair was evaluated using the following factors: protein matching and interaction matching (Hao et al., 2014). In the integration process, the network with a closer genetic relationship to *L. vannamei* was considered as the target network, whereas the one to be integrated was designated the query network. When the proteins in the target and query network are homologous to each other and to the same unigene in *L. vannamei*, the score of this protein was 2. When the homologous proteins in the target and query networks were homologous to different unigenes of *L. vannamei*, the score of the protein was 1. Finally, if a protein in the target/query network was not homologous to any protein in the query/target network, the score of this protein in the integrated network was 0. Interaction scores, that is edge matching, were determined similarly. When a protein interaction pair existed in both the target and query networks, its score was 3, whereas interactions solely from the target or query network were scored 2 and 1, respectively. Interaction from the target network has higher score than that from the query network because that the target network had a closer genetic relationship with *L. vannamei*. The final score of an interaction pair was the sum of protein scores and interactions scores after six iterations of integration. Therefore, the maximum and minimum score of an interaction pair were 42 and 1, respectively.

### Functional Annotation of Unclassified Proteins and Unigenes

First, functions of proteins in the PIN were assigned to Gene Ontology (GO) categories. The GO database (Ashburner et al., 2000) provides a standardized annotation of protein and gene attributes integrating species and databases, including molecular functions, biological processes, and cellular components. We used the biological process category as the functional annotation of proteins. GO annotation can be shown as a hierarchical diagram based on the relationships of GO terms. The GO items in each GO level in the hierarchical structure were downloaded from the GO database. Proteins and unigenes without GO annotations were considered as unclassified proteins and unigenes. The functions of unclassified proteins and unigenes were annotated using the method from our previous method (Hao et al., 2014) based on the function(s) of neighbor proteins in networks.

## Results and Discussion

### Construction of the *L. vannamei* PIN

Through transcriptome sequencing of *L. vannamei* haemocytes, the sequences of 52,073 unigenes were obtained (Xue et al., 2013). A total of 13937, 12810, 20517, 22668, 22941, 20457, and 6692 proteins were obtained from the STRING database for *D. melanogaster*, *A. gambiae*, *C. elegans*, *M. musculus*, *R. norvegicus*, *H. sapiens*, and *S. cerevisiae*, respectively. The number of proteins and interaction pairs of the seven model organisms from the STRING databases are shown in Table 1.

**Table 1:** Protein dataset from the STRING databases.

Organism	Protein	Interacting pairs
*D. melanogaster*	13937	7963454
*A. gambiae*	12810	3265176
*C. elegans*	20517	5778268
*M. musculus*	22668	12614042
*R. norvegicus*	22941	13433196
*H. sapiens*	20457	11353056
*S. cerevisiae*	6692	2007134

For constructing the PIN of *L. vannamei*, the seven model organism based protein–protein interaction sub-networks should be firstly constructed. These sub-networks were obtained according to the alignment of the unigene sequence in *L. vannamei* and the protein sequences in the model organisms. Thus, seven model organism based protein–protein interaction sub-networks were constructed. The features of these sub-networks are shown in Table 2. The largest proportion of interactions were inferred from the *D. melanogaster* based sub-network because of the closer genetic relationship between *L. vannamei* and *D. melanogaster*, whereas most protein–protein interactions were inferred from *H. sapiens* due to the complexity of the interaction system in *H. sapiens*.

**Table 2:** Features of model organism-based protein–protein interaction sub-networks.

Organism	Protein–protein interaction sub-network^a^		
	Unigene	Protein	Protein–protein interaction
*D. melanogaster*	7155 (64.3%)	3437 (24.66%)	56330 (1.73%)
*A. gambiae*	5695 (50.43%)	2779 (21.69%)	18931 (0.33%)
*C. elegans*	3483 (61.22%)	2034 (9.91%)	23132 (0.29%)
*M. musculus*	7156 (60.81%)	3801 (16.77%)	49131 (0.43%)
*R. norvegicus*	6639 (56.99%)	3650 (15.91%)	41107 (0.33%)
*H. sapiens*	7940 (67.19%)	4221 (20.63%)	74523 (0.55%)
*S. cerevisiae*	3506 (83.34%)	1659 (24.79%)	20444 (1.02%)

*^a^Numbers in brackets show the proportions of related unigenes, proteins, and interactions in the sub-network accounting for the global dataset of the model organism.*

The *L. vannamei* PIN was obtained by integrating the seven model organism based sub-networks. The global alignment-based integration method was used in a six-round integration to construct the *L. vannamei* PIN. The scale of the resulting network in each round of integration is shown in Table 3. More than half of the proteins, unigenes and interactions in the final integrated PIN come from the *D. melanogaster* based sub-network. The final *L. vannamei* PIN is composed of 4,858 proteins and 104,187 interactions (Supplementary Tables S1, S2).

**Table 3:** The scale of the integrated network after each round of data integration.

	First round	Second round	Third round	Fourth round	Fifth round	Sixth round
Matching node (n1)	2522	1852	3420	3598	3843	1560
Node in query network (n2)	2779	2034	3801	3650	4221	1659
n1/n2	90.75%	91.05%	89.98%	98.58%	89.68%	94.03%
Matching edge (m1)	16797	19014	35172	38282	55398	14748
Edge in query network (m2)	18931	23132	49131	41107	74523	20444
m1/m2	88.83%	82.20%	71.59%	93.13%	74.34%	72.14%
Final node	3694	3897	4332	4399	4701	4858
Final edge	58464	62582	76541	79366	98491	100776

### Scoring of the *L. vannamei* PIN

**FIGURE 1:**
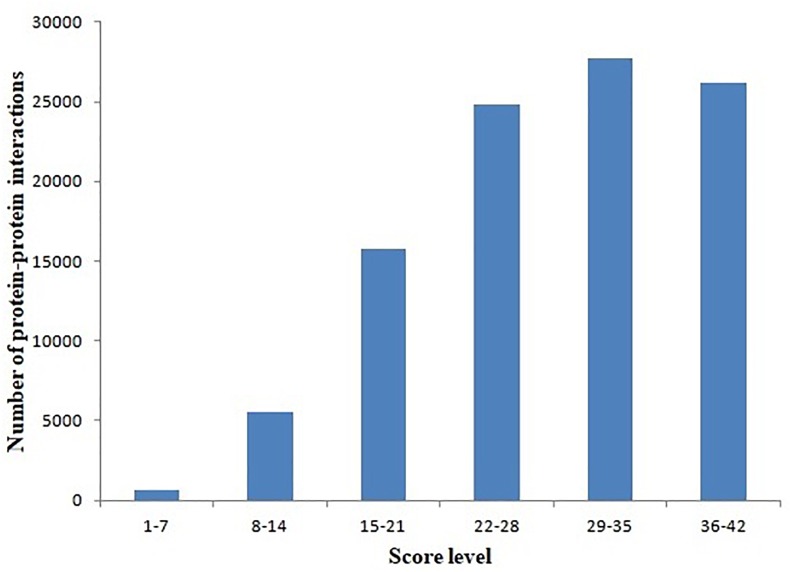
Distribution of protein–protein interactions at different score ranges. The scores of protein–protein interactions were dived into six periods from 1 to 42. The period 29–35 include most interactions.

Protein–protein interactions were inferred from different model organisms. To characterize the confidence of the inferred interactions, the interactions in the *L. vannamei* PIN were scored (see section “Materials and Methods”). The score is mainly determined by two factors: protein matching and interaction matching. The distribution of protein–protein interactions with different score ranges is shown in Figure 1. Interactions were centralized in the 22–28, 29–35, and 36–42 score ranges. The 29–35 range includes the most of the interactions (27,739), accounting for 27.53% of all the interactions in the PIN. The high percentage of interactions inferred from *H. sapiens* (about 73.96%) is the most important contribution for this distribution. The large amount of high-confidence nodes and edges led to high final scores for many interactions.

**FIGURE 2:**
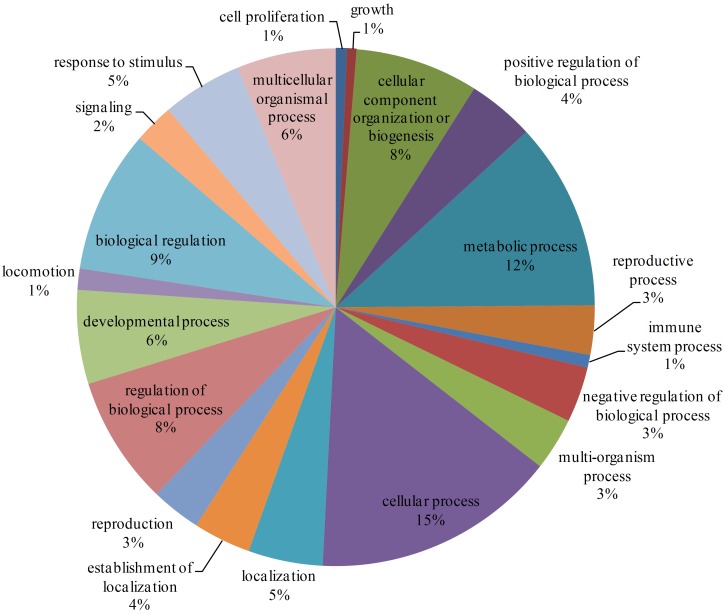
GO enrichment analysis of proteins with interaction scores ≥40. Different GO annotations were represented by different colors. The GO annotations were the children items of “Biological process” in the GO database.

There are 7905 interactions with scores ≥40 in the network. With the GO enrichment analysis, most proteins were function in regulation (17%, including biological regulation and regulation of biological process), cellular process (15%) and metabolic process (12%) (Figure 2). Fbxo6, Rnf4, Fbxo22, Fbxo21, Fbxl18, Det1, and Vhl constituted the core proteins of the sub-network composed by these high-score interactions. These proteins are all related with the ubiquitin ligase complex, with Fbxo6, Fbxo22, Fbxo21 as the substrate-recognition component of the SCF-type E3 ubiquitin ligase complex and Vhl as a target recruitment subunit in the E3 ubiquitin ligase complex. Ubiquitination is an important posttranslational protein modification, regulating a host of critical cellular processes. The E3 ubiquitin ligase complex is the central player in the ubiquitination process and plays a decisive role in the specificity of substrate ubiquitination (Li et al., 2018). The discovery of this sub-network constituted by these proteins might indicate the existence of ubiquitin ligase complex in *L. vannamei*.

### Immune-Specific Sub-Network of *L. vannamei*

**FIGURE 3:**
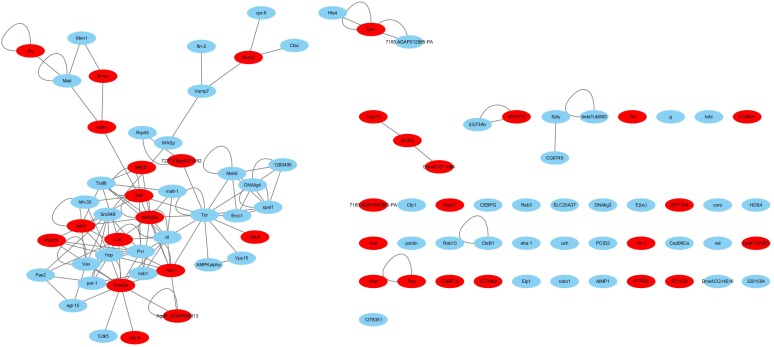
The immune-specific sub-network of *L. vannamei*. Proteins in signaling pathways are shown with red color.

Haemocytes are important immune tissues in *L. vannamei*. Many immune-related proteins exist in haemocytes and play significant roles in the immune system through their interactions. To further investigate the immune system of *L. vannamei*, proteins in the hierarchical branch of the “immune system process (GO:0002376)” and the interactions among these proteins were collected from the global PIN to generate the immune-specific sub-network. In total, 93 proteins were annotated as immune-related proteins and 115 interactions were included in the immune-specific sub-network, including a large connected group of 108 interactions composed of 48 proteins (Supplementary Table S3 and Figure 3). In the immune-specific sub-network 36 proteins (38.7%) were annotated to signaling pathways, including Ras, Rap1, MAPK, NF-kappa B, HIF-1, Pi3K-Akt, Jak-STAT, mTOR, Wnt, etc., supporting the close relationship between signaling transduction processes and immune mechanisms. The existence of the Jak-STAT signaling pathway was verified by Chen et al. (2008) and shown to participate the shrimp immune process. These authors reported a full-length cDNA sequence for a STAT protein from *Penaeus monodon* and demonstrated the effect of the WSSV virus on activating the Jak-STAT signaling pathway. Furthermore, the proteins Src42A in the PI3K-Akt signaling pathway, IKKbeta in the NF-kappa B signaling pathway, Akt1 in the Rap1 signaling pathway, Tor in the Glycerophospholipid metabolism pathway and dl in the Glutathione metabolism closely interacted with many other immune-related proteins in different signaling pathways, indicating their possible important coordinating roles in the immune system.

### Evolution of the *L. vannamei* Immune Specific Sub Network

**FIGURE 4:**
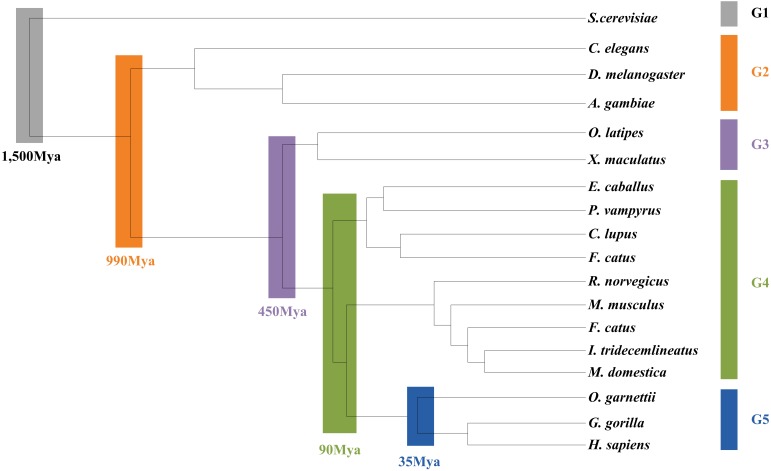
Age groups classification of *L. vannamei* proteins. Different age groups were represented by different colors. Age groups were classified according to NCBI Taxonomy and literature. Age groups reflect the approximate evolution stage of proteins. The length of branches is not correlated to evolution time.

**FIGURE 5:**
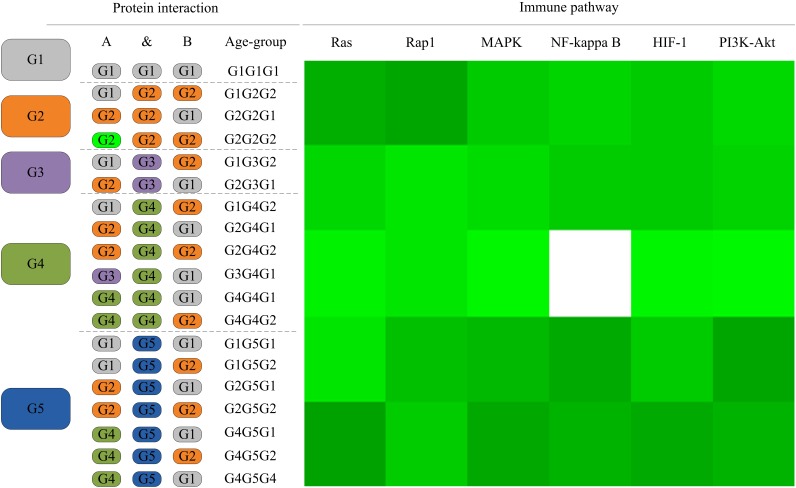
The distribution of protein interactions in different age groups in the six immune pathways studies in this work. Protein interactions in the six signaling pathways in this study were divided into different “age groups” according to the origins of the corresponding components. A blank on the right side represents a lack of protein origins in this age group. For each pathway, the proportions of interactions in each age group of evolution to all interactions in this pathway are shown in different shades of green. A darker shade of green signifies a larger proportion.

As the *L. vannamei* immune-specific sub-network was generated from model organisms located in different evolutionary branches, we compared the *L. vannamei* immune sub-network with 18 organisms and investigated the original organisms as well as the preferred evolutionary paths of the *L. vannamei* immune sub-network. The selected organisms include the seven model organisms used in the network construction and the other 11 organisms with relatively more orthologous to the 93 proteins in the immune-specific sub-network. Based on a study by Chen C.Y. et al. (2014), the 18 species were classified to five age groups (G1–G5) according to the evolution branches (Figure 4): Based on this division, we categorized each protein interaction in the *L. vannamei* immune-specific sub-network. For each protein interaction, the origin of an interaction and its two related proteins were defined separately using the procedure similar to Li et al. (2012). A protein/interaction was assigned to the earliest age group according to its organism sources. For example, if a protein/interaction exists in a G1 group organism, it is assigned to G1 group. If a protein/interaction exists in a G2 group organism but not in a G1 group organism, it is assigned to G2 group. If a protein/interaction exists only in G5 group organism(s), it is assigned to G5 group. Finally, the origin of a protein–protein interaction (including an interaction and two proteins) was assigned to the evolutionary age group in which the last component of the interaction appeared. The origins of six signaling pathways with more than ten proteins (Ras signaling pathway, Rap1 signaling pathway, MAPK signaling pathway, NF-kappa B signaling pathway, HIF-1 signaling pathway, and PI3K-Akt signaling pathway) in the sub-network were further analyzed (Figure 5). Although most of the proteins in the six signaling pathways origin in G1 and G2 groups, the interactions were appeared in different age groups in these pathways. Most interactions in Rap1 pathways originated from G1 group, and most interactions in the MAPK, NF-kappa B and PI3K-Akt pathways were found at G4 and G5 group. Most interactions in the Ras and HIF-1 pathways were found in G5 group. These results indicate that the different signaling pathways may arise and mature in different ages.

**FIGURE 6:**
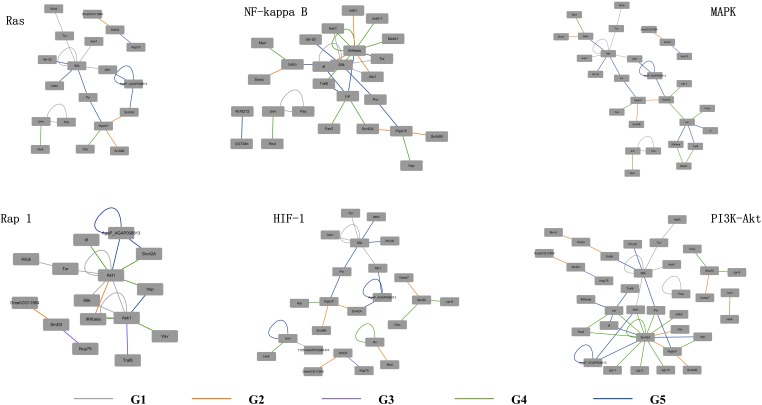
Evolutionary origins of the six signaling pathways. The evolution age groups of interactions in the signaling pathways were shown in different colors, with gray representing G1, orange representing G2, purple representing G3, green representing G4, and blue representing G5.

The detailed evolutionary origins of each pathway were shown in Figure 6. In the Ras signaling pathway, interactions between Ask1 and S6k originate in G1 group. Interaction between Ptp61F and hop originates in G4 group. The route from Ask1 to hop was completed until the interactions from S6k to Ptp61F were formed in G5 group. However, in the G4 group, the direct interaction between proteins Ask1 and hop emerged in Rap1 signaling pathway. The same situation happened to proteins Ask1 and Src42A, whose interaction route were completed through S6k-Pur-Ptp61F until G5 group in Ras signaling pathway but directly interact in the G4 group in Rap1 signaling pathway. Proteins hop and Src42A are both tyrosine protein kinase which play roles in lots of biological process such as defense response to virus/bacterium, compound eye development, peptidyl-tyrosine autophosphorylation and so on. In addition, proteins hop and Src42A have similar molecular functions such as transferase activity and ATP binding. The similar evolutionary history and functions as well as the similar interacted proteins of protein hop and Src42A may indicate that the function of proteins may have relationship with their evolutionary history.

### Functional Assignment of Unclassified Proteins and Unigenes

**FIGURE 7:**
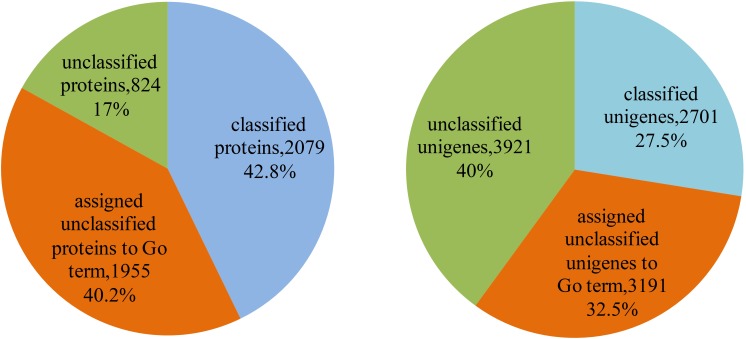
Function annotation of proteins and unigenes in *L. vannamei*. The left pie chart shows the results of function annotation of proteins, and the right pie chart represents the results of function annotation of unigenes.

The 4,858 proteins in *L. vannamei* PIN correspond to 9,813 unigenes, in which the function of 2,079 proteins and 2,701 unigenes were annotated according to GO annotation, whereas the other 2,779 proteins and 7,112 unigenes are still unclassified, accounting for 57.2% of all proteins and 72.5% of all unigenes in the PIN. The function of unclassified proteins and unigenes was annotated using the method based on the neighbor proteins in the network. Finally, the functions of 1,955 proteins and 3,191 unigenes were annotated, which account for approximately 70.3 and 44.9% of the unclassified proteins and unigenes, respectively (Supplementary Table S4). With this annotation, unclassified protein and unigenes in the PIN decreased to 17 and 40%, respectively (Figure 7).

**FIGURE 8:**
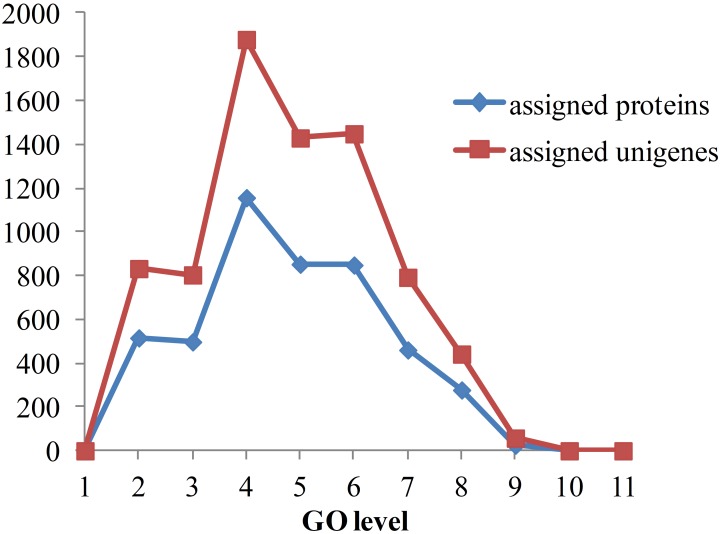
Distribution of annotated proteins and unigenes with depths of GO terms. Depths of GO terms were identified according to the hierarchical structure of GO terms in Gene Ontology database. Assigned proteins in this work were shown in blue line and assigned unigenes were shown in red line.

As the functional annotation was distributed in different GO levels (which are organized in a hierarchical structure), we further analyzed the distribution of newly classified proteins and unigenes in different GO levels. As shown in Figure 8, level 4 contains the most of the annotated proteins and unigenes. A total of 79 proteins were annotated to have immune-related functions, including innate immune response, regulation of innate immune response and antifungal humoral response. Multi GO terms were annotated to many protein and unigenes because protein/unigenes may have several functions. Of the 79 immune-related proteins, 39 were annotated with certain KEGG pathways, in which 13 were in signaling-related pathways, 9 were in metabolic pathway and 8 were in endocrine-related pathways. It indicates that immune system is closely related to the signaling process, metabolic process and endocrine system. Although the exact functions of these newly annotated proteins/unigenes still need to be further validated, the prediction of functions provides effective candidates for the recognition of targets and mechanistic analysis for *in vivo* experiments.

## Conclusion

With the development of systems biology, a systematic view has been widely applied to various aspects of biological research and significantly enhances scientific studies. In this work, we constructed the first PIN for *L. vannamei* haemocytes based on the transcriptome sequencing data and the protein interactome of seven model organisms. The PIN provides a global view of the interactome in *L. vannamei* haemocytes and a platform for the study of the functional sub-networks. Many signaling pathways were identified in the immune-specific sub-network constructed from the global PIN. By analyzing of the evolution of six signaling pathways, we found the differences in their evolutionary origin and speculated that the function for some proteins might have relationship with their evolutionary process. Furthermore, the functional annotation of 1,955 unclassified proteins offers new references for the protein function investigation. This is the first large-scale PIN of shrimp, which supplies a necessary platform and tool for the study of *L. vannamei* immune and regulation mechanisms, as well as provides an important systems blueprint for the exploration of proteome and interactome for other hydrobios.

## Ethics Statement

The study was approved by College of Life Sciences, Tianjin Normal University.

## Author Contributions

LZ and DW performed the experiments. EW and JS contributed in the guideline and revision of the manuscript. TH designed the experiments. TH, BW, and XF wrote the manuscript.

## Conflict of Interest Statement

The authors declare that the research was conducted in the absence of any commercial or financial relationships that could be construed as a potential conflict of interest.

## Supplementary Material

TABLE S1 The interactions in *L.vannamei* PIN.

TABLE S2 The cytoscape file for *L.vannamei* PIN.

TABLE S3 The interactions in immune-specific sub-network of *L.vannamei*.

TABLE S4 Functional assignment of unclassified proteins and unigenes.
